# Expression of *Sonic Hedgehog* and pathway components in the embryonic mouse head: anatomical relationships between regulators of positive and negative feedback

**DOI:** 10.1186/s13104-021-05714-5

**Published:** 2021-08-05

**Authors:** Crystal L. Sigulinsky, Xiaodong Li, Edward M. Levine

**Affiliations:** 1 Department of Ophthalmology and Visual Sciences, John A. Moran Eye Center, University of Utah, Salt Lake City, UT USA; 2 Department of Ophthalmology and Visual Sciences, Vanderbilt Eye Institute, Vanderbilt University Medical Center, 1161 21st Ave S, B3307 MCN/2569, Nashville, TN 37232 USA; 3 Department of Cell and Developmental Biology, Vanderbilt University, Nashville, TN USA

**Keywords:** Sonic hedgehog, Gli1, Patched, Hedgehog interacting protein, Gene expression, Retina, Palatal rugae, Hair follicle, Eyelid, Molar, Embryonic day 15.5

## Abstract

**Objective:**

The Hedgehog pathway is a fundamental signaling pathway in organogenesis. The expression patterns of the ligand *Sonic Hedgehog* (*Shh*) and key pathway components have been studied in many tissues but direct spatial comparisons across tissues with different cell compositions and structural organization are not common and could reveal tissue-specific differences in pathway dynamics.

**Results:**

We directly compared the expression characteristics of *Shh,* and four genes with functional roles in signaling and whose expression levels serve as readouts of pathway activity in multiple tissues of the embryonic mouse head at embryonic day 15.5 by serial in situ hybridization. The four readout genes were the positive feedback regulator *Gli1,* and three negative feedback regulators, *Patched1*, *Patched2*, and *Hedgehog Interacting Protein*. While the relative abundance of *Gli1* was similar across tissues, the relative expression levels and spatial distribution of *Shh* and the negative feedback regulators differed, suggesting that feedback regulation of hedgehog signaling is context dependent. This comparative analysis offers insight into how consistent pathway activity could be achieved in tissues with different morphologies and characteristics of ligand expression.

**Supplementary Information:**

The online version contains supplementary material available at 10.1186/s13104-021-05714-5.

## Introduction

**Fig. 1:**
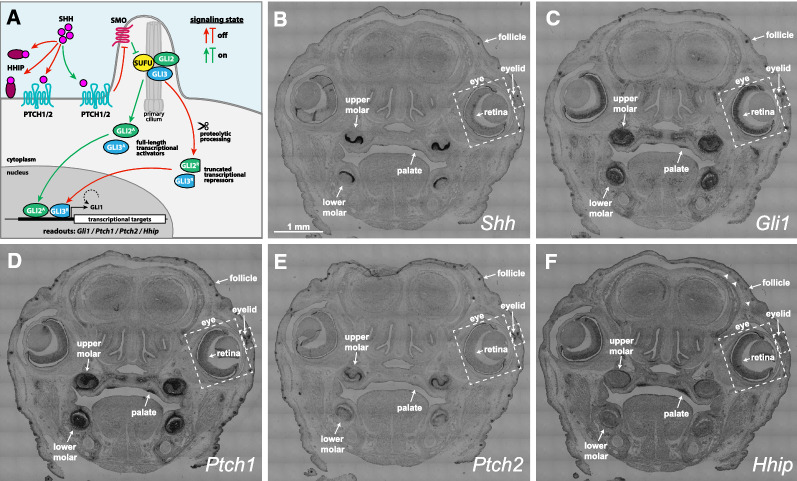
Expression patterns for Shh and Hh pathway components in developing organs of the embryonic mouse head. **A** Simplified schematic of Hh signaling. See "Introduction" section for details. **B**–**F** in situ hybridizations for *Shh* (**B**), *Gli1* (**C**), *Ptch1* (**D**), *Ptch2* (**E**), and *Hhip* (**F**) in adjacent coronal sections of the mouse at E15.5. Arrowheads in F denote additional hair follicles

Shh is a secreted glycoprotein belonging to the Hedgehog (Hh) family of intercellular signaling molecules. The mechanics of Hh signaling is complex, extending from ligand production through signal transduction to the cell- and tissue-specific responses (reviewed in [1–4]). In its simplest iteration (Fig. 1A), binding of Shh to its receptor, Patched 1 (Ptch1) or, in some cases, Ptch2, relieves inhibition of the G-protein coupled receptor Smoothened (Smo). Activated Smo inhibits proteolytic processing of the GLI transcriptional effectors Gli2 or Gli3 into truncated repressor forms through destabilization of complexes between Gli2 or Gli3 and Suppressor of Fused (Sufu). The resulting accumulation of full-length GLI proteins in the nucleus promotes the expression of Hh target genes.

Transcriptional targets of the pathway not only mediate cellular responses to Hh ligands, but also participate in feedback loops that further regulate pathway activity. The principal positive feedback loop involves the transcriptional effector Gli1. *Gli1* expression is activated in response to Gli2 transcriptional activation [5–7]. This, together with its activator function, allows Gli1 to increase signaling levels while retaining dependence on active Hh signaling. *Gli1* expression is therefore an excellent indicator of pathway activity.

Ptch1, Ptch2, and Hhip participate in negative feedback that act at the level of Hh reception [8–10]. *Ptch1* transcripts are also upregulated in response to Hh signaling [11–13], and evaluation of phenotypes and Hh pathway activity in *Ptch1* mutant mice shows that Hh activity is sensitive to *Ptch1* gene dosage [14–16]. In addition to Smo inhibition, upregulation of *Ptch1* (*Ptc* in *Drosophila*) also sequesters Hh ligands and desensitizes the cell to Hh signal [17]. *Patch2* shares sequence homology with *Ptch1*, binds Hh ligands with high affinity and inhibits Shh-induced changes in gene expression [18, 19]. *Ptch2* is also upregulated in response to Hh signaling, but this can be context dependent [18–20]. Additionally, Ptch2 fails to block changes in gene expression induced by a constitutively active form of Smo and is unable to replace Ptch1 function in *Ptch1* mutant basal carcinoma cells but does preserve some ligand dependent signaling in *Ptch1-*null fibroblasts [19, 21, 22]. Interestingly, Ptch1 and 2 can non-autonomously inhibit Smo, possibly through secretion of a cholesterol precursor [10]. Like *Ptch1* and *Ptch2*, *Hhip* is upregulated in response to Hh signaling. Hhip also binds Hh ligands with high affinity and can attenuate Hh signaling through ligand sequestration [8, 9, 23, 24]. Like Ptch1 and Ptch2, Hhip also negatively regulates the level of Hh ligands to which the responding cell is exposed.

In our studies on Hh signaling in retinal neurogenesis in mice, which begins at ~ E11.5, we’ve observed that *Shh* expression can be difficult to detect even though Hh signaling has essential functions in retinal development, and *Shh*, expressed in retinal ganglion cells (RGCs), is the sole Hh ligand employed during this time (reviewed in [25]). We asked, if *Shh* expression was lower in the retina than in other anatomical structures, how would *Gli1* and the expression of the negative feedback regulators compare? And if differences in expression exist across structures, could anatomical differences correlate with how *Shh* and the feedback components are expressed? To address these questions, we performed in situ hybridizations for *Shh, Gli1, Ptch1, Ptch2,* and *Hhip* on serial sections of an E15.5 embryonic mouse head. Direct comparisons were made for the 5 genes across 6 tissues with active sonic hedgehog signaling.

## Main text

### Materials and methods

#### Animals

129SvImj mice (stock #2448, Jackson Laboratory, Bar Harbor, ME) were bred overnight and pregnant dams at gestational day 15.5 were euthanized with a Euthanex EP-1305 CO_2_ delivery system following AALAC guidelines. Upon removal from uteri, embryos were rapidly euthanized by decapitation with surgical scissors and heads were placed into Hanks Buffered Saline Solution (HBSS) supplemented with 20 mM HEPES and 6 mg/ml glucose at room temperature.

#### In situ* hybridization*

Heads were fixed overnight at 4 °C in 4% formaldehyde in PBS pH7.5, 2 mM EGTA, followed by cryoprotection with 20% sucrose/PBS, and frozen in OCT. 12 µm serial sections were stained with digoxigenin-labeled anti-sense probes produced by in vitro transcription of sequence-verified linearized plasmids (Additional file 1: Figure S1). Section in situ hybridization was performed as previously described [26–28].

#### Sample size and data collection

Five embryos from three separate litters were analyzed. The images shown are from a single animal. Data collection was by visual assessment from two unblinded but independent observers (CLS, EML).

#### Image capture

Sections were imaged at 10X magnification on a Leica DMR microscope under brightfield illumination. Image tiles (8-bit, 1388 × 1036 pixel) were acquired with a QICAM Fast 1394 (QImaging, Burnaby, Canada) and automated scanning stage (Märzhäuser, Wetzlar, Germany). Mosaic images were assembled using a Syncroscan montaging system (Synoptics, Frederick, MD). Close up views of the hair follicles were imaged at 20X magnification with a Spot-RT camera (Diagnostic Instruments, Sterling Heights, MI) on a Nikon E-600 microscope using differential interference contrast. Due to their small size, hair follicles could not be analyzed for all probes on adjacent serial sections; similar positions within representative morphologically matched follicles were imaged. Figures were assembled with Photoshop and Illustrator CC (Adobe, San Jose, CA).

### Results

**Fig. 2:**
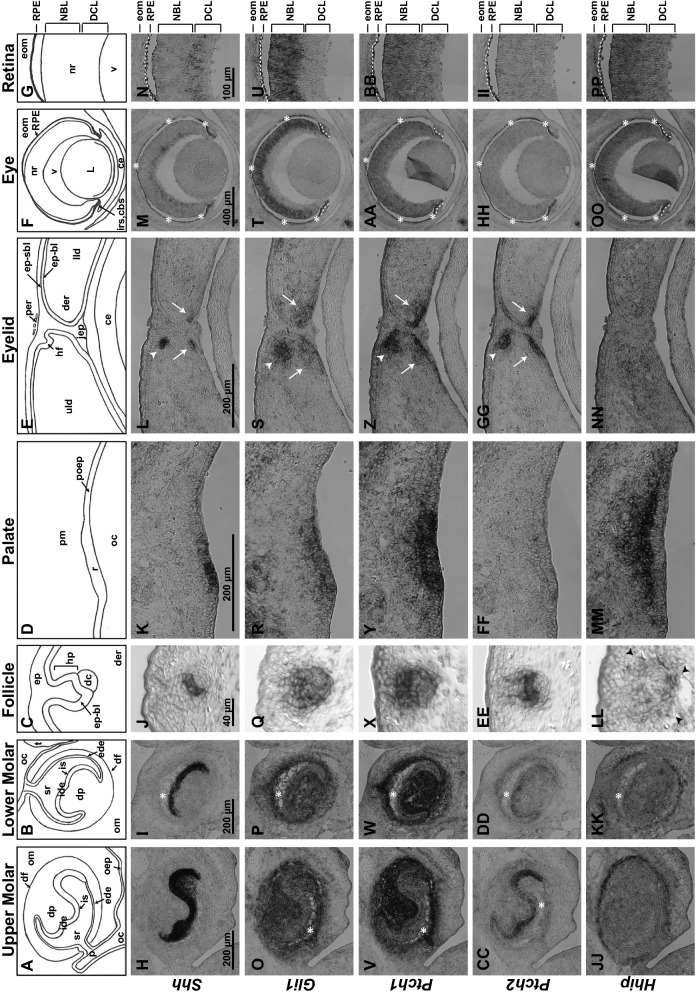
Close up comparisons of expression patterns. **A**, **B** Schematics of upper (**A**) and lower (**B**) molars with buccal side to the left, lingual side to the right. **C**–**G** Schematics of stage 3 hair follicle (**C**), palate (**D**) eyelid (**E**), eye (**F**), and retina (**G**). **H**–**PP**) Expression patterns of *Shh* (**H**–**N**), *Gli1* (**O**–**U**), *Ptch1* (**V**–**BB**), *Ptch2* (**CC**–**II**), and *Hhip* (**JJ**–**PP**) in each structure. See abbreviations list for descriptions. White asterisks indicate histological artifacts where tissues are lacking. The asterisks for the eye structures also indicate the pigmentation of the RPE and is not mRNA staining. Black arrowheads in **LL** point to *Hhip* in the condensing mesenchyme surrounding the follicle. White arrows in eyelid panels point to the eyelid signaling field, and the arrowhead denotes a hair follicle. White dashed lines in eye panels denote the iris stroma (below line). White dashed lines in retina panels denote the RPE (below lines) and extraocular mesenchyme including the condensing scleral mesenchyme (above lines)

Figure 1 shows the expression patterns of *Shh*, *Gli1 Ptch1*, *Ptch2*, and *Hhip* in the context of the head. We identified upper and lower molars, palatal rugae, retina, eyelid, and hair follicles as tissues for comparison based on *Shh* and *Gli1* expression. *Shh* expression identified the cellular sources of Hh signal and was most readily detected in the molars (Fig. 1B). Despite the small sizes of the hair follicles, palatal rugae, and eyelid, *Shh* expression was still evident at this scale. In contrast, the retina exhibited a low level of *Shh* expression that was disproportionate to its relatively large size. *Gli1* expression, the indicator of Hh signaling, was similarly robust across all 6 tissues (Fig. 1C). *Ptch1* expression was also robust in all 6 tissues (Fig. 1D) although its expression in the retina appeared lower by comparison to the levels of *Gli1* in each tissue. This is easier observed in Fig. 2. The patterns of *Ptch2* were most similar to *Shh,* although expression levels in the retina and palatal rugae were too low to assess at this scale (Fig. 1E). *Hhip* was detected in the molars, hair follicle, palatal rugae, and retina; expression in the eyelid was too low to assess. Interestingly, *Hhip* was abundant in the retina and palatal rugae (Fig. 1F), where *Ptch2* was lowest.

Figure 2 shows the expression patterns at scales appropriate for each tissue. Illustrations for each structure are presented (Fig. 2A-G), with specific anatomical and gene expression descriptions provided in the supplement. (Additional file 2). As above, our focus here is to compare gene expression patterns across the structures.

*Shh* was generally restricted to epithelial tissues within the molars, hair follicles, palatal rugae, and eyelids (Fig. 2H–L). The retina is primarily composed of cells from the neuroepithelium but *Shh* expression was similarly segregated, in this case, to the differentiated cell layer (DCL) where the RGCs are located (Fig. 2M, N). By and large, *Shh* expression was robust relative to the size of the tissue except in the retina, where expression was disproportionately lower.

*Gli1* and *Ptch1* exhibited largely overlapping patterns of expression (Fig. 2O–AA). Both were expressed throughout epithelial and mesenchymal tissues. Interestingly, mesenchymal tissues stained more strongly for *Gli1* and *Ptch1* in the molars and hair follicles (Fig. 2O–Q, V–X), while epithelial staining was stronger in the palate and eyelids (Fig. 2R, S, Y, Z). In the neural retina, *Gli1* expression overlapped with that of *Ptch1* in the neuroblast layer (NBL; Fig. 2T, U, AA, BB). *Gli1* was not detected in the DCL whereas *Ptch1* extended into the DCL.

*Ptch2* overlapped with *Shh* but was expressed more broadly (Fig. 2CC–EE, GG), consistent with earlier reports [20, 29]. Two exceptions are the palatal rugae and retina where *Ptch2* expression was not detected (Fig. 2FF, HH, II). Since *Ptch2* is reliably detected in the retina by transcriptomic and RT-PCR based methods ([28], personal observation (XL and EML), the lack of detection here suggests low and potentially broad expression.

*Hhip* was expressed in a narrow band in the mesenchyme surrounding the molars at the outer edge of *Ptch1* and *Gli1* expression, at a distance from *Shh*-expressing cells (Fig. 2JJ, KK). Although not as distinct as in the molars, *Hhip* was expressed in the condensing mesenchyme surrounding the epithelial compartment of the hair follicle (Fig. 2LL; arrowheads in Fig. 1F denote additional follicles). *Hhip* expression within the palate exhibited a graded and robust pattern that was strongest in the palatal mesenchyme (pm) immediately adjacent to the *Shh*-expressing ruga (r). Expression in the eyelid was too low to assess (Fig. 2NN). As with *Ptch1*, *Hhip* expression in the retina extended across both the NBL and DCL in a graded manner that was strongest in the NBL.

### Discussion

Through direct comparative analysis, the expression patterns of several feedback regulators of Hh signaling were assessed. To first address the question that motivated this study, we found that the abundance of *Shh* mRNA is comparatively low in the retina, but pathway activity, as assessed by *Gli1* expression, is robust and on par with other tissues. This suggests tissue-specific differences in how robust signaling is achieved, and selective utilization of the negative feedback factors is one possibility. Supporting this, we observed nonoverlapping expression of *Ptch2* and *Hhip,* even in structures that express both. Thus, in addition to providing a mechanism to prevent overactive signaling, the utilization of specific feedback inhibitors could contribute to more efficient Hh signaling at lower levels of ligand expression.

Of the three negative regulators, the expression pattern for *Ptch1* was most similar to *Gli1*. Although this makes it the least likely to have a tissue-selective role, it does make it the most reliable of the negative regulators to mark the field of active signaling. This is not surprising since Ptch1 is required in the majority of tissues for ligand-dependent signaling [14]. Subtle differences, however, in its expression levels whether quantitative or spatial, or in the localization or modification of Ptch1 protein, could contribute to tissue-specific influences on signaling [30].

*Ptch2* and *Hhip*, however, exhibited unique expression characteristics. In the molars and hair follicles, their expression domains marked the two ends of the signaling field, with *Ptch2* closest to the source of ligand and *Hhip* expressed at the outermost extent of signaling. Only *Ptch2* was detected in the eyelid and only *Hhip* in the palate and retina. Although *Ptch2* and *Hhip* are both negative feedback regulators and act at the level of ligand availability, their differential utilization could account for differences in signaling efficiency across structures. For example, if the retina is most efficient at Hh signaling as suggested, could *Hhip* have a role in this? How this might occur is not clear but there are differences in how Ptch2 and Hhip regulate ligand availability. Whereas both are on the cell membrane where they bind and remove Shh ligand by endocytosis, Hhip is also secreted and sequesters ligand extracellularly. This could keep ligand intact, releasing it for signaling at a later time or in another location. Thus, Hhip could also have a supportive role in Hh signaling.

### Conclusions

This study describes the spatial expression patterns of *Shh*, *Gli1*, *Ptch1*, *Ptch2* and *Hhip* in 6 anatomical structures. The patterns of *Gli1* and *Ptch1* suggest similar levels of signaling across structures with different levels of *Shh* expression. The patterns of *Ptch2* and *Hhip* suggest different roles in controlling the level of signaling in each tissue.

## Limitations

Colorimetric detection is qualitative and does not allow for precise measurements of mRNA expression levels. Another limitation is that one developmental stage was assessed and temporal differences in gene expression could exist across anatomical structures. However, hedgehog signaling is active before E15.5 in the tissues analyzed so differences due to asynchronous pathway initiation across tissues is unlikely. Another limitation is the indirect nature of using gene expression as indicators of ligand availability or signaling activity. Determining whether differences in *Ptch2* and *Hhip* utilization contribute to qualitatively similar levels of *Gli1* expression and pathway activity requires functional perturbations and evaluation of ligand availability for each structure.

## Supplementary Information

**Additional file 1**:** Figure S1**. In situ mRNA hybridization probe templates. cDNA inserts were sequenced from each end, aligned with BlastN, and mapped onto their respective NCBI reference sequence using NCBI's Sequence Viewer 3.24.0 (https://www.ncbi.nlm.nih.gov/tools/sviewer/).

**Additional file 2**: Anatomical descriptions of each structure and gene expression patterns.

## Data Availability

The plasmids and datasets used and/or analyzed for the current study are available from the corresponding author on reasonable request.

## Molars

ide: Internal dental epithelium
ede: External dental epithelium
is: Intermediate stratum
sr: Stellate reticulum
dp: Dental papilla
df: Dental follicle
p: Pedicle
oc: Oral cavity
om: Oral mesenchyme
oep: Oral epithelium
t: Tongue

## Hair follicles

ep: Epidermis
ep-bl: Basal layer of epidermis
hp: Hair peg
dc: Dermal condensate
der: Dermis

## Palatal rugae

r: Palatal rugae
poep: Palatal oral epithelium
pm: Palatal mesenchyme
oc: Oral cavity

## Eyelid

uld: Upper lid
lld: Lower lid
ep-sbl: Suprabasal layer of epidermis
ep-bl: Basal layer of epidermis
der: Dermis
jep: Junctional epithelium
per: Residual periderm
hf: Hair follicle
ce: Corneal epithelium

## Eye and retina

nr: Neural retina
RPE: Retinal pigmented epithelium
v: Vitreous
L: Lens
ce: Corneal epithelium
pom: Periocular mesenchyme
irs: Iris stroma
cbs: Ciliary body stroma
NBL: Neuroblast layer
DCL: Differentiated cell layer
eom: Extraocular mesenchyme
