# Supplementary material for: Expression of Sonic Hedgehog and pathway components in the embryonic mouse head: anatomical relationships between regulators of positive and negative feedback
Source: BMC Res Notes. 2021 Aug 5;14:300. doi: 10.1186/s13104-021-05714-5 (PMC8340441; doi:10.1186/s13104-021-05714-5)
Supplement: Supplementary file 1 — Additional file 1: Figure S1. In situ mRNA hybridization probe templates. cDNA inserts were sequenced from each end, aligned with BlastN, and mapped onto their respective NCBI reference sequence using NCBI's Sequence Viewer 3.24.0 (https://www.ncbi.nlm.nih.gov/tools/sviewer/). [file 13104_2021_5714_MOESM1_ESM.pdf]

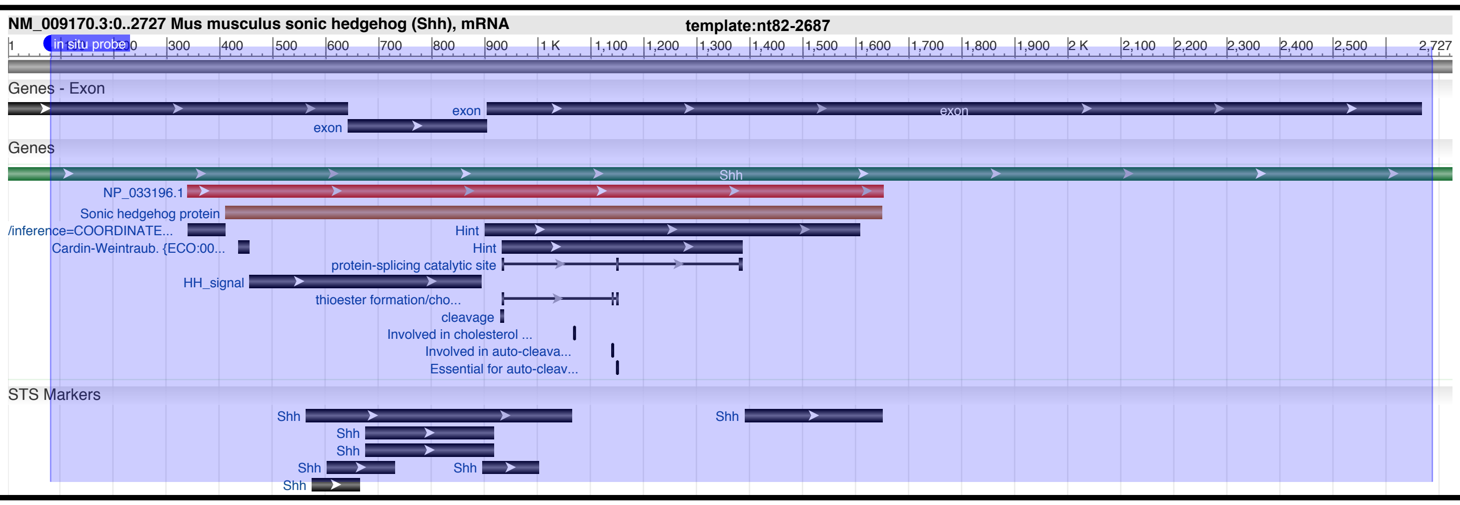
NM_009170.3:0..2727 Mus musculus sonic hedgehog (Shh), mRNA
template:nt82-2687
in situ probe
Genes - Exon
exon
exon
exon
Genes
Shh
NP_033196.1
Sonic hedgehog protein
/inference=COORDINATE...
Hint
Cardin-Weintraub. {ECO:00...
Hint
protein-splicing catalytic site
HH_signal
thioester formation/cho...
cleavage
Involved in cholesterol ...
Involved in auto-cleava...
Essential for auto-cleav...
STS Markers
Shh
Shh
Shh
Shh
Shh
Shh
Shh

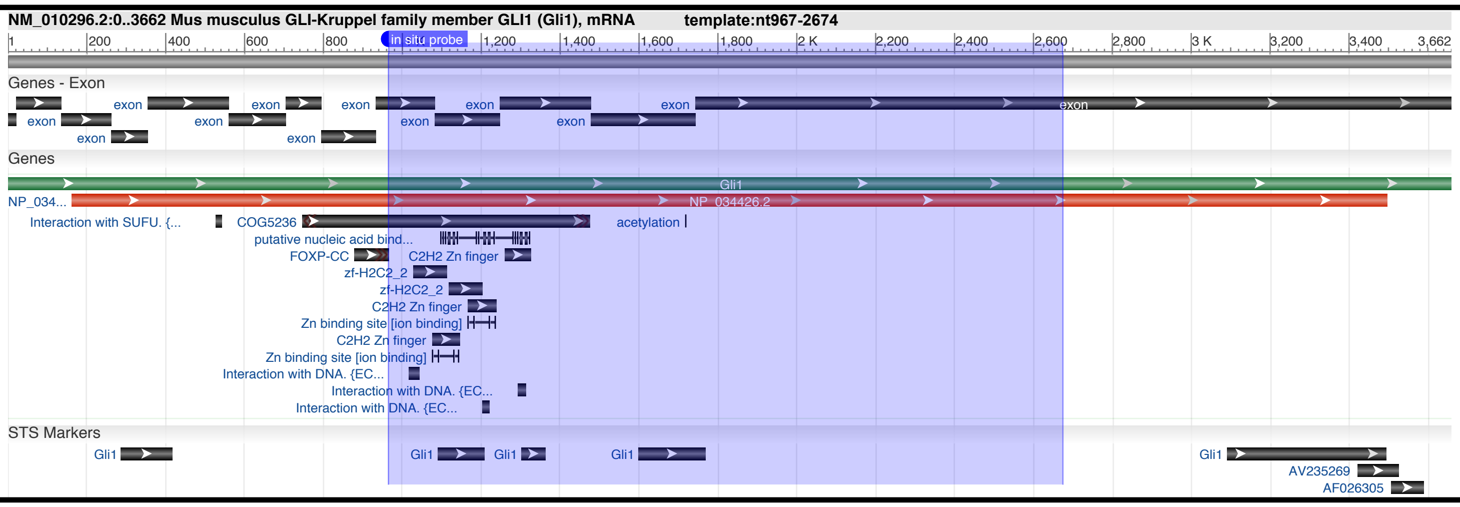
NM_010296.2:0..3662 Mus musculus GLI-Kruppel family member GLI1 (Gli1), mRNA
template:nt967-2674
in situ probe
Genes - Exon
exon
Genes
Gli1
NP_034...
NP_034426.2
Interaction with SUFU. {...
COG5236
acetylation
putative nucleic acid bind...
FOXP-CC
C2H2 Zn finger
zf-H2C2_2
zf-H2C2_2
C2H2 Zn finger
Zn binding site [ion binding]
C2H2 Zn finger
Zn binding site [ion binding]
Interaction with DNA. {EC...
Interaction with DNA. {EC...
Interaction with DNA. {EC...
STS Markers
Gli1
Gli1
Gli1
Gli1
Gli1
AV235269
AF026305

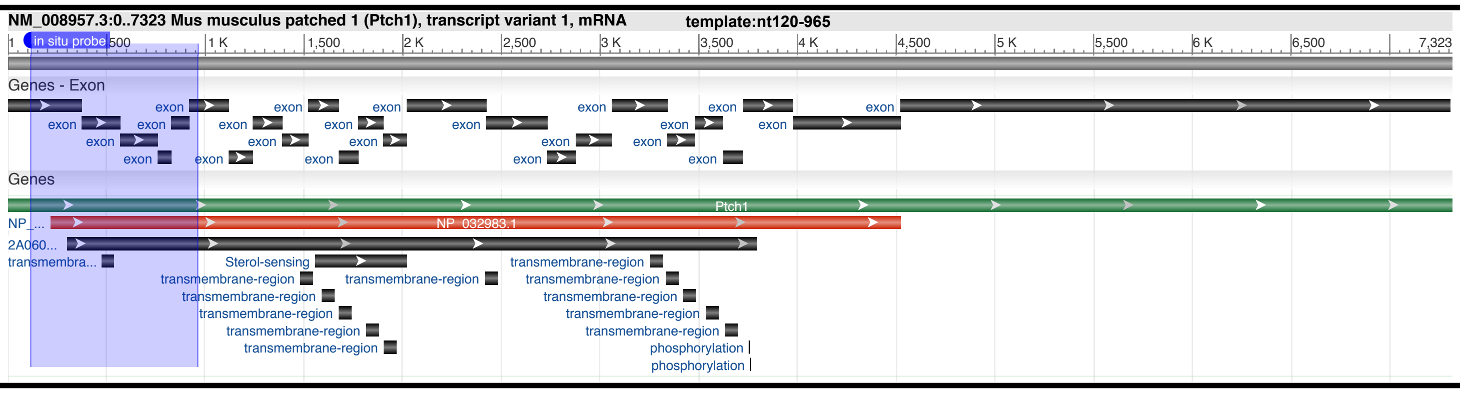
NM_008957.3:0..7323 Mus musculus patched 1 (Ptch1), transcript variant 1, mRNA
template:nt120-965
in situ probe
Genes - Exon
exon
Genes
Ptch1
NP_...
NP_032983.1
2A060...
transmembra...
Sterol-sensing
transmembrane-region
phosphorylation
phosphorylation

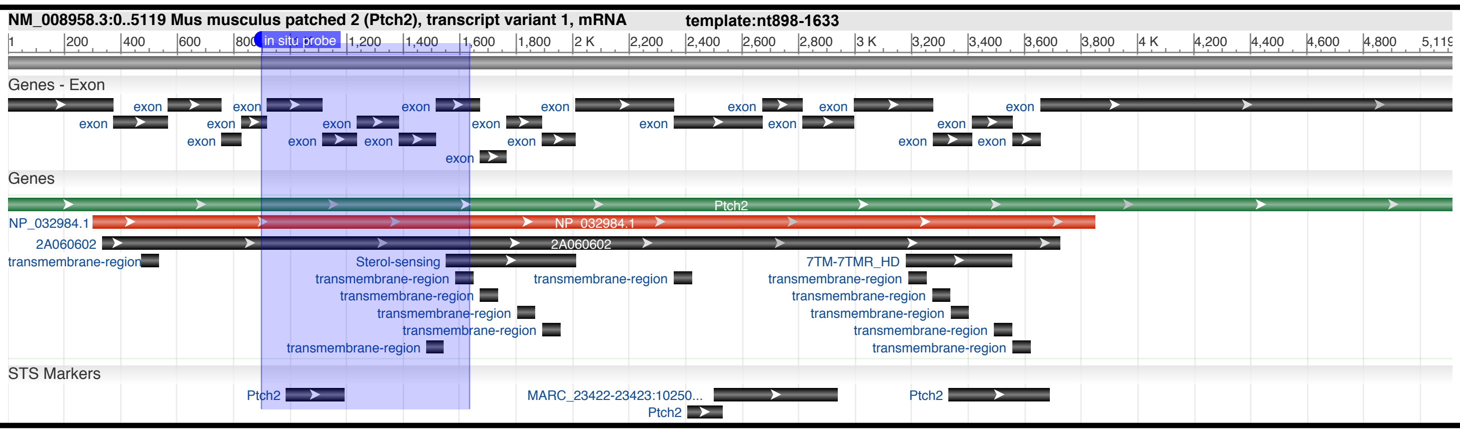
NM_008958.3:0..5119 Mus musculus patched 2 (Ptch2), transcript variant 1, mRNA
template:nt898-1633
in situ probe
Genes - Exon
exon
Genes
Ptch2
NP_032984.1
NP_032984.1
2A060602
2A060602
transmembrane-region
Sterol-sensing
7TM-7TMR_HD
STS Markers
Ptch2
MARC_23422-23423:10250...
Ptch2
Ptch2

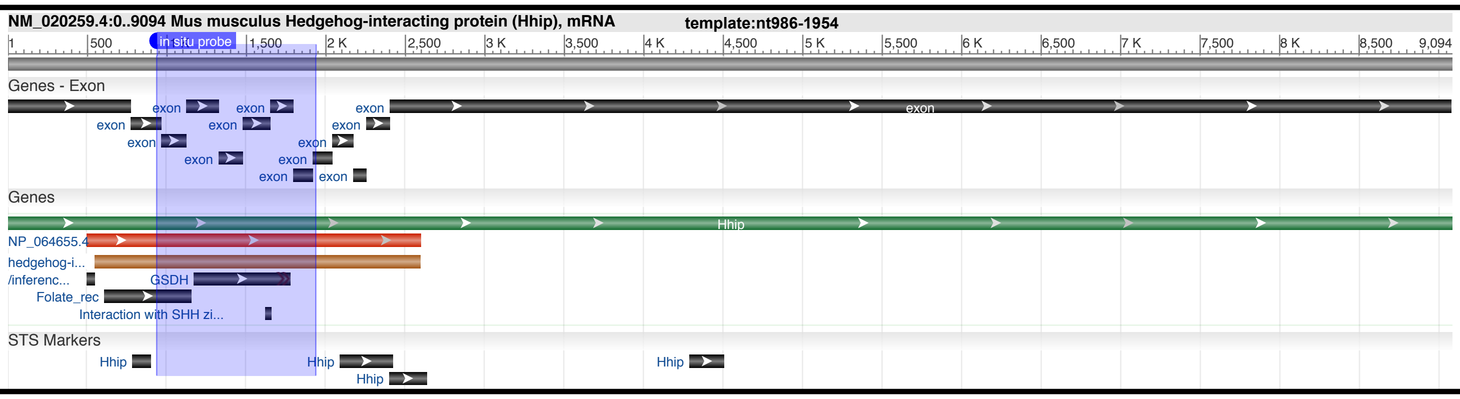
NM_020259.4:0..9094 Mus musculus Hedgehog-interacting protein (Hhip), mRNA
template:nt986-1954
in situ probe
Genes - Exon
exon
Genes
Hhip
NP_064655.4
hedgehog-i...
/inferenc...
GSDH
Folate_rec
Interaction with SHH zi...
STS Markers
Hhip
Hhip
Hhip
Hhip
